# Study of a two species microbial community by an inferential comparative genomic analysis tool: Spatial Analytical Microbial Imaging

**DOI:** 10.1016/j.mex.2015.06.004

**Published:** 2015-06-25

**Authors:** Pei Zhang, Paloma Valverde, Douglas Daniel, Peter Fox

**Affiliations:** a Department of Civil, Environmental & Sustainable Engineering, Arizona State University, Arizona State University, PO Box 5306, Tempe, AZ 85287, USA; b Biodesign Institute at Arizona State University, 1001 S. McAllister Ave., PO Box 875001, Tempe, AZ 85287-5710, USA; c Department of Sciences, Wentworth Institute of Technology, 550 Huntington Avenue, Boston, MA 02115, USA

**Keywords:** Spatial Analytical Microbial Imaging, Microbial community analysis, Co-culture, Genomic copy number, 3D imaging analysis, 3D locus, Inferential comparative genomic copy number, Spatiotemporal microbial community

## Abstract

Most molecular fingerprinting techniques, including denaturing gradient gel electrophoresis (DGGE) [1], comparative genomic hybridization (CGH) [2], real-time polymerase chain reaction (RT-PCR) [3], destroy community structure and/or cellular integrity, therefore lost the info. of the spatial locus and the *in situ* genomic copy number of the cells. An alternative technique, fluorescence *in situ* hybridization (FISH) doesn't require sample disintegration but needs to develop specific markers and doesn't provide info. related to genomic copy number.

Here, a microbial analysis tool, Spatial Analytical Microbial Imaging (SAMI), is described. An application was performed with a mixture of *Synechocystis sp.* PCC 6803 and *E. coli* K-12 MG1655. The intrinsic property of their genome, reflected by the average fluorescence intensity (AFI), distinguished them in 3D. And their growth rates were inferred by comparing the total genomic fluorescence binding area (GFA) with that of the pure culture standards. A 93% of accuracy in differentiating the species was achieved.

• SAMI does not require sample disintegration and preserves the community spatial structure.
• It measures the 3D locus of cells within the mixture and may differentiate them according to the property of their genome.
• It allows assessment of the growth rate of the cells within the mixture by comparing their genomic copy number with that of the pure culture standards.

## Graphical abstract

**Figure:**
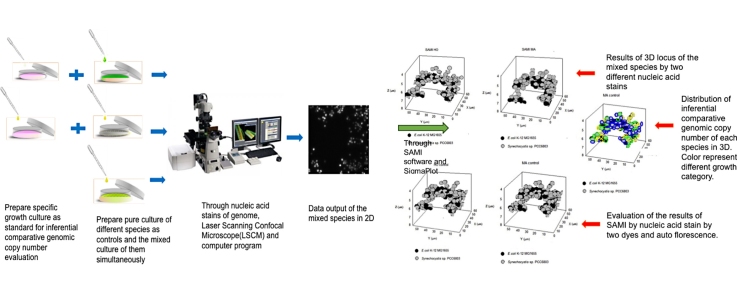

## Method

A microbial analytical tool, SAMI, was developed for spatial-temporal analysis of a pure or a mixed culture microbial community. This method maintains microbial community spatial structure and can be used to identify 3D locus, differentiate species and evaluate *in situ* relative growth rate of individual cells within the community through comparative genomic copy number and inferential statistics. A comparison of SAMI to some existing methods is shown in Table 1. A pre-test on the pure culture can be done samely by SAMI first to exam if it may be applicable for the mixture. A time-course profile of the mixed population can be generated through temporal monitoring of the community by SAMI without sample fixation, hence providing a more comprehensive and in depth understanding of a two species microbial community.

## Recommended equipment

• Carl Zeiss Confocal Laser Scan Microscope (CLSM 510 Meta)
• LSM 510 software (http://www.softpedia.com/get/Multimedia/Graphic/Graphic-Viewers/Zeiss-LSM-Image-Browser.shtml)
• SAMI software (*via* http://www.bioechem.com)
• SigmaPlot 10.0.1 (http://www.sigmaplot.com/products/sigmaplot/produpdates/prod-updates3.php)

## Sample preparation, imaging and analysis

### Culture preparation

#### *Escherichia coli* K-12 MG 1655 pure culture

To prevent contamination, *E. coli* K-12 MG1655 was initially transformed by inserting ampicillin resistant genes (AmpR) through electroporation [4]. The size of the ampicillin resistant gene was approximately 1.25 kb [5], which is not significant in comparison to the whole genome size of *E. coli* K-12 MG 1655. To obtain a pure culture, a single colony of *E. coli* K-12 MG 1655 was grown in 25 ml LB solution supplemented with 25 μl of 200 μg/μl ampicillin sodium salt (69523, Sigma) and incubated at 37 °C for 12 h.

#### *E. coli* K-12 MG 1655 pure culture standard

One milliliter of *E. coli* K-12 MG 1655 pure culture was transferred onto an alcohol sterilized glass slide, which was placed in a petri-dish at room temperature (27 °C ± 1 °C). Two milliliters of freshly made BG-11 media were added daily onto the glass slide for three days to support *E. coli* K-12 MG1655 pure culture growth on the glass surface.

#### *Synechocystis* sp*.* PCC 6803 pure culture

One colony of *Synechocystis sp.* PCC 6803 growing on an BG-11 agar plate was grown in 25 ml BG-11 media at room temperature (27 °C ± 1 °C) to allow *Synechocystis sp.* PCC 6803 to grow in suspension for more than two weeks.

#### *Synechocystis sp.* PCC 6803 pure culture standard

One ml of *Synechocystis sp*. PCC 6803 pure culture was transferred onto an alcohol sterilized glass slide, and then placed in a petri-dish at room temperature (27 °C ± 1 °C). Two ml of BG-11 media was added on the glass slide every day for three days to allow the growth of the *Synechocystis sp*. PCC 6803 pure culture on the glass surface.

#### Mixed culture preparation

One milliliter of pure culture *E. coli* K-12 MG1655 suspension was added onto the *Synechocystis sp.* PCC 6803 pure culture grown on a glass slide to allow the growth of both species as phase I. Two milliliters of BG-11 media was then added to the glass slide every day for three days as phase 2, and the mixture was allowed to grow at room temperature (27 °C ± 1 °C) for another three days as phase 3. Pure culture standards of *Synechocystis sp.* and *E. coli* K-12 MG1655, were prepared concurrently to serve as controls.

#### Preparation of slow growth culture standards

To obtain a slow growth culture standard of *E. coli* K-12 MG1655, a single colony of *E. coli* K-12 MG1655 was isolated from the LB agar plate and transferred to 10 ml M9 media. The culture was then incubated in a shaker at 27 °C ± 1 °C for 3 days. One ml of the solution were then transferred into 10 ml of fresh M9 media and grown under the same conditions for 3 more days. Serial dilutions were repeated for a minimum of 3 times in order to obtain a slow growth culture standard.

To obtain a slow growth culture standard of *Synechocystis sp.* PCC 6803, a single colony of *Synechocystis sp.* PCC 6803 was grown in 10 ml of BG-11 media for 3 days under room light and room temperature (27 °C ± 1 °C). One ml was then transferred into 10 ml of fresh BG-11 media and cells allowed to grow for 3 more days. Serial dilutions were repeated for a minimum of 3 times.

Inferential statistical analysis was used to obtain genomic copy number standard of the slow growth cells. An absolute genomic copy number of the slow growth pure culture can be quantified by real-time PCR or an spectroscopic method [3]. It has been previously reported that *Synechocystis sp.* PCC 6803 motile wild type has about 60 genomes per cell in stationary phase and this number varies for different strains [6]. The genome of *Synechocystis sp.* PCC 6803 slow growth pure culture was first labeled and inferential statistics were used in comparative genomic analysis to obtain comparative genomic copy number of each *Synechocystis sp.* PCC 6803 in the mixture.

## Sample fixation, stain and preparation for imaging

### Fixation

Because the nucleic acid dyes utilized in this study were membrane-permeable, samples could be fixed for one point sampling or processed without fixation for sequential sampling. The temporal observation of samples was performed without fixation. When fixation was utilized, samples were washed twice with buffer and then fixed for 3 h in glass slides [7]. The glass slides were placed on a sterilized petri-dish and immersed in 1 ml of 4% buffered paraformaldehyde solution for 3 h at room temperature. The buffered solution was made from stock paraformaldehyde (16% paraformaldehyde, CAS #30525-89-4, Electron Microscopy Sciences) in 2 M NaCl and 0.1 M PBS buffer (15 mM MgCl2; 0.8 g sodium chloride, 0.2 g potassium chloride, 1000 ml sterile distilled water, pH 7.5) [8].

2.2 Fluorescent dye staining

A mixture of BisBenzimide H 33258 (HO) and Mithramycin A (MA) solution dyes (1 μg/ml each) was used to stain all samples. Samples were washed once with 1X Tris Buffer (5 mM Tris–HCl, pH 7.6, 8 mM NaCl) before staining. HO dye solution (DNA Quantitation Kit, DNA-Q, Sigma) and MA solution (M6891Mithramycin A, Sigma) were prepared following the manufacturer’s recommendations. The final concentration of MgCl_2_ in the dye mixture was 15 mM at the time of measurement [9]. The dye solution was set under dark conditions at room temperature for 20 min to reach equilibrium. Then 1 ml of the mixed dye working solution was added to each sample for 20 min. The quantification of DNA has been reported to require a high salt concentration[9]. For peak fluorescence, at least 200 mM NaCl is required for purified DNA and 2.0–3.0 M for crude samples. Mg^2+^ ions have no effect on the assay in the final concentration range of 0.5–0.1 M and the salt concentrations of up to 3 M NaCl will not affect the assay negatively.

3. Confocal microscopy preparation and imaging

For visible light and high numerical aperture objectives (>0.8) a pixel size of ∼0.1–0.2 μm is recommended [10]. The pixel size of the CLSM system was optimized to 0.116 μm. In order to process fast dynamic scans, sequential raster scan was used and the scan speed was optimized at 0.9 μs/pixel to reduce delays between acquisitions. The other parameters were optimized and set up accordingly by using LSM 510 software. These parameters were as follows: Amplifier offset at 0.1; Amplifier gain at 1; Power of 405 nm at 0.5 mW; Pinhole at 0.58 Airy equivalent; Optical slice 0.6 μm; Frame size at 512 μm × 512 μm; Interval at 0.1 μm.

The emission spectra of HO, MA and autofluorescence are 460 nm, 560 nm and 725 nm respectively. Therefore, the dyes were discriminated from each other and autofluorescence using coated filters. Images were collected in different tracks. MA was set at track 1 with a bandpass filter at 505–570 nm, and HO was set at track 2 with a bandpass filter at 420–480 nm. Both HO and MA were set with the mirror reflection. Autofluorescence of *Synechocystis* sp*.* PCC 6803 was excited by Argon/2 at 488 nm with a different channel and a longpass filter 650. The emitted light then hits the secondary dichroic (NFT 545) which reflect light of wavelength lower than 545 nm and transmit light longer than 650 nm.

## Image analysis

**Fig. 1:**
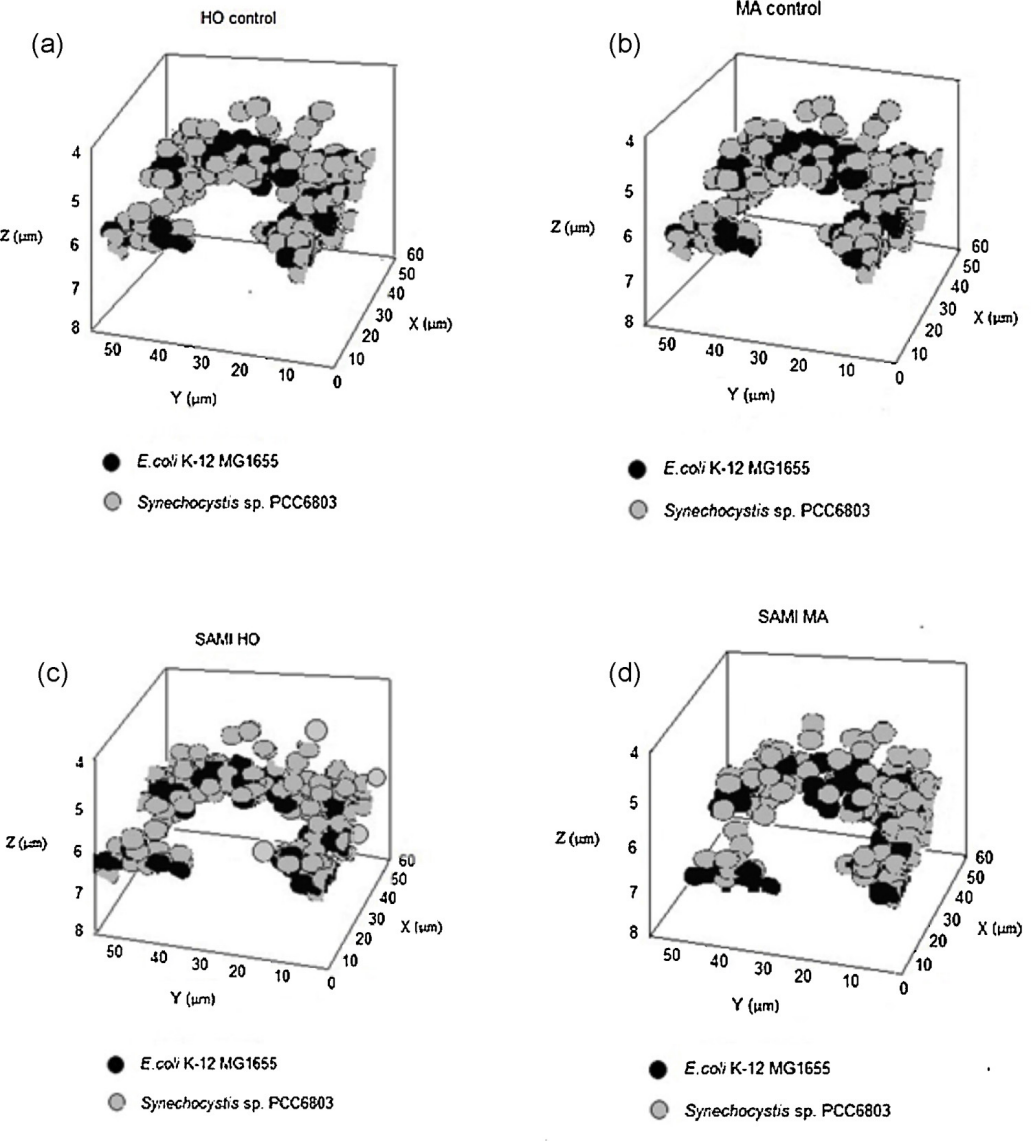
The controls and SAMI spatial distribution of the two species. (a), (b) The controls represent the true spatial distribution of the two species in the sample by utilizing autofluorescence emitted by *Synechocystis* sp. PCC 6803. HO and MA stain the nucleic acid of all species. (c), (d) The spatial distribution of the two species *via* HO and MA nucleic acid staining using the SAMI method.

**Fig. 2:**
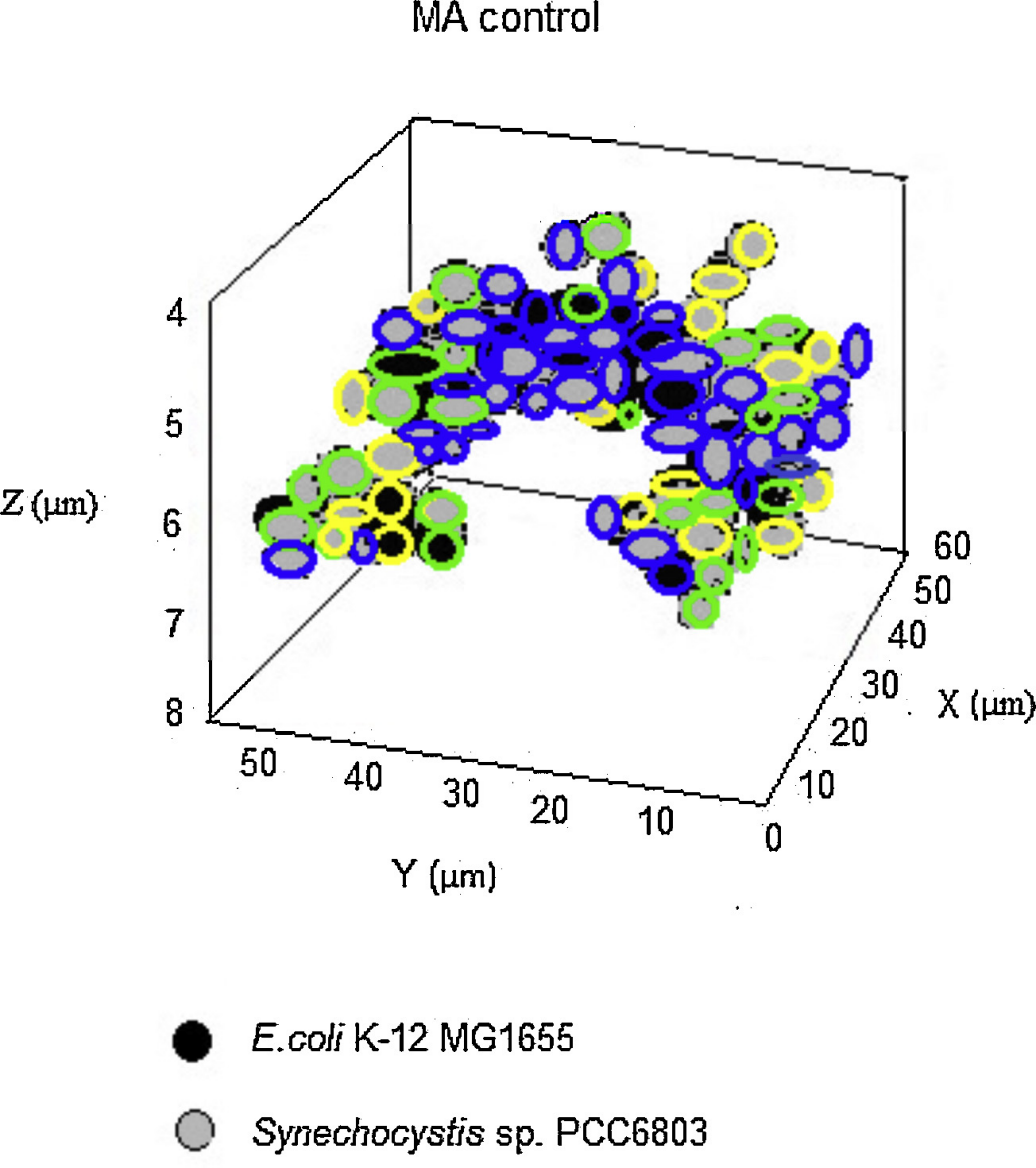
3D distribution of relative genomic copy number in the mixture of *E*. *coli* K-12 MG1655 and *Synechocystis* sp. PCC 6803. Blue circles, represent the relatively faster-growth cells, which have larger number of genomic copies than that of the growth standard. Green circles, represent equal-growth cells with equal number of genomic copies as the growth standard. Yellow circles, represent the slower-growth cells with less genomic copy than that of the growth standard. The growth standard can be the culture of oligoploidy, merodiploidy or polyploidy *etc*. (For interpretation of the references to color in this figure legend, the reader is referred to the web version of this article.)

1 In both 2D and 3D data analysis, channels were split, thresholds were set to enclose most data point in a narrow range. The values, such as mean value, geometric center, binding area, were analyzed based on the original imaging data acquired by LSM 510 software and the results displayed.
2 Do the same process to mixed samples and concurrently grown pure cultures and the slow growth pure cultures.
3 Compare the average fluorescence intensity (AFI) of each cell in mixed culture to the mean value of the pure culture and differentiate the cells in the mixed culture.
4 Plot identified results using 3D data (*x*, *y*, *z*) in sigmaplot 10.0 and compare with the control (Fig. 1).
5 Compare the GFA of the identified cells in the mixed culture with that of the slow growth pure culture standard to evaluate the relative genomic copy number of each cell and identify their 3D locus in the mixture (Fig. 2).

**Fig. 3:**
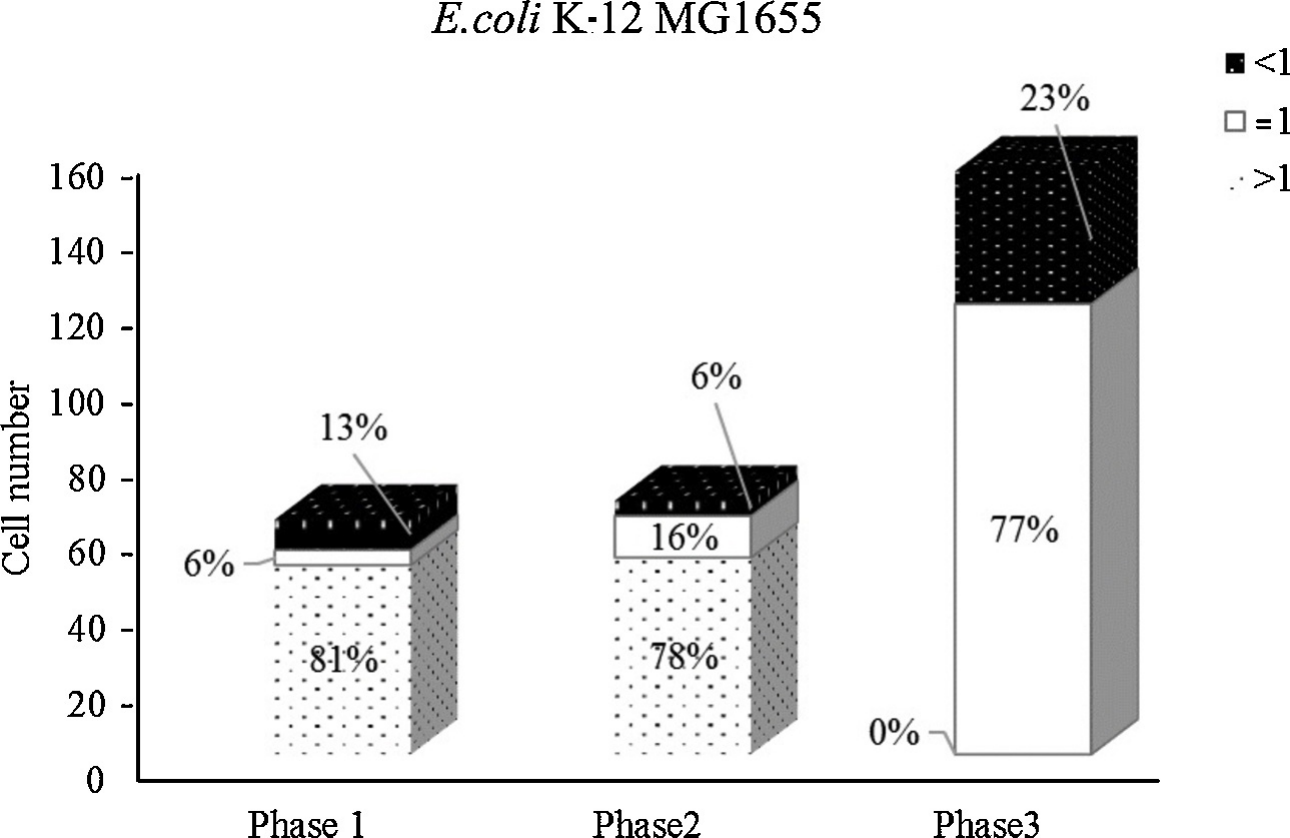
Percentage distribution of inferential comparative genomic copy number of *E. coli* K-12 MG1655 in the mixed culture at different time courses as explained in 1.5. The genomic copy number of each cell in the sample is evaluated by comparing its genome size with that of the growth standard with the genomic copy number considered as “1”. The black bars represent the cells with lower genomic copy number than that of the standard, indicating slower-growth cells. The white bars represent the cells with equal genomic copy number as the standard, hence indicating equal growth rate. The doted bars represent the cells with higher genomic copy number than the standard, demonstrating faster-growth cells. The time interval between each phase is three days.

*E. coli* K-12 MG1655 maintains the similar amount of cells in the mixed community during the first two phases (Fig. 3). The total number of *E. coli* K-12 MG1655 drastically increased (doubled) in phase 3 (Fig. 3). However, the analysis of the inferential comparative genomic copy number shows that cells are more actively dividing at phases 1 and 2 than at phase 3. Maybe due to the lack of nutrients, most of the cells in phase 3 were relatively slow dividers, which indicates a temporal shifting of the population composition in the near future (Fig. 3).

**Fig. 4:**
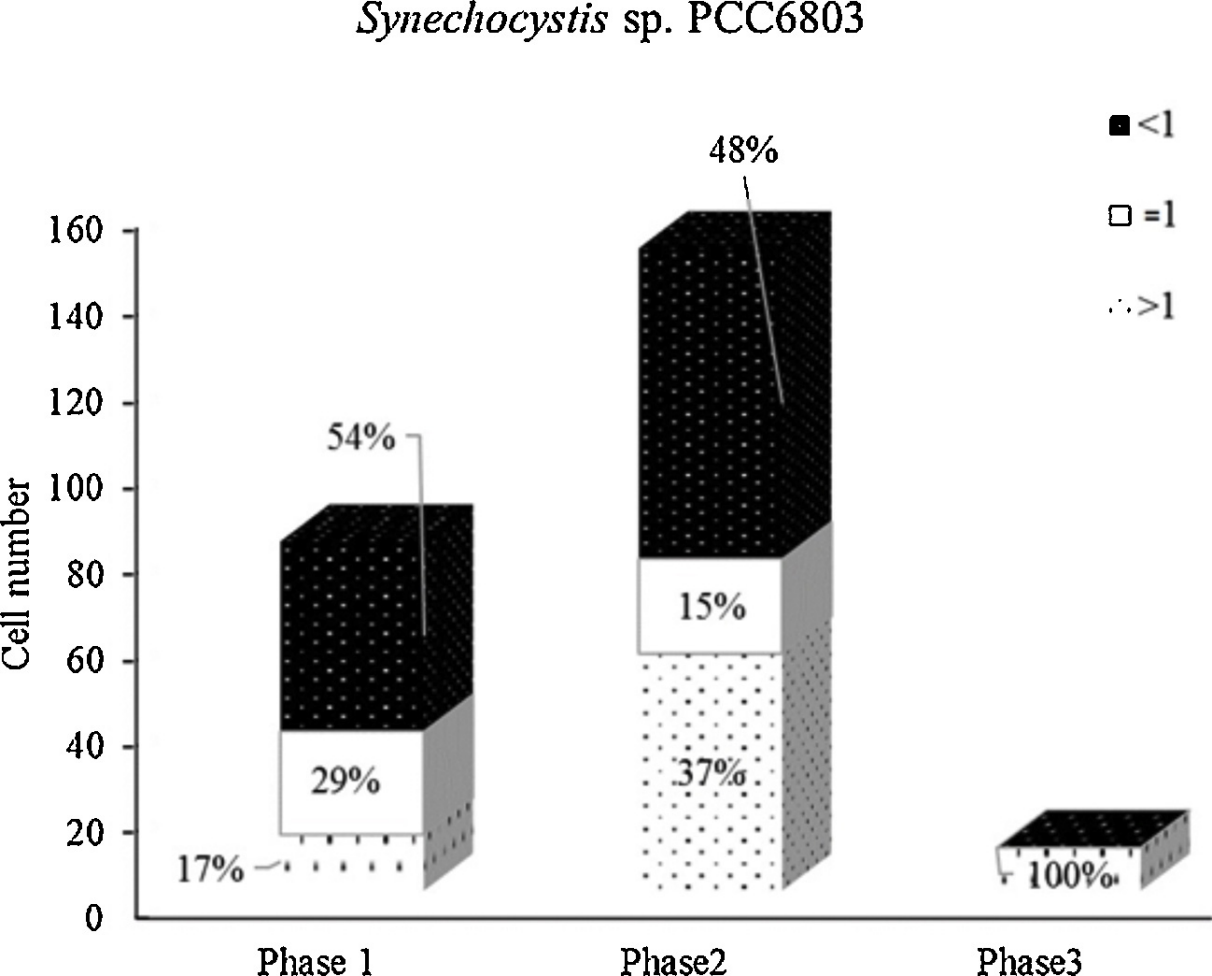
Percentage distribution of inferential comparative genomic copy number of *Synechocystis* sp. PCC 6803 in the mixed culture at different time courses. *Synechocystis* sp. PCC 6803 in this case is closely linked with the cell division cycle and results in substantial losses from the population. The genomic copy number of each cell in the sample is evaluated by comparing the genome size with that of the growth standard with the genomic copy number considered as “1”. The black bars represent the cells with lower genomic copy number than that of the standard, indicating slower-growth cells. The white bars represent the cells with equal genome copy number and growth rate as the standard. The doted bars represent the cells with higher genomic copy number than that of the standard, hence indicating a faster growth rate than the standard. The time interval between each phase was three days.

During phase 3 the cell number of cyanobacteria was dramatically reduced but the genomic copy number was higher than that of the standard indicating a faster growth (Fig. 4). These results suggest an adaptive role of cyanobacteria as previously shown by others [11], [12]. Cell death is observed when environmental stress exceeds cell tolerance and in some situations it has been categorized as programmed cell death (PCD). Thus, an adaptive role has been suggested for cyanobacteria PCD whereby the death of some individuals optimizes the probability of population persistence [11], [12].

## Method validation

In this assay, the autofluorescence of *Synechocystis* sp*.* strain PCC 6803 was concurrently recorded to verify the accuracy of SAMI in differentiating species. The autofluorescent image overlapped the outline of *Synechocystis* sp*.* strain PCC 6803 in the mixed culture, hence distinguishing them from *E. coli* K-12 MG1655. The autofluorescent overlaying images validated the accuracy of SAMI (Fig. 1(a) and (b)). The resulting 3D Images from the SAMI analyses are shown (Fig. 1(c) and (d)). The autofluorescence property of *Synechocystis* sp*.* Strain PCC 6803 was utilized to validate SAMI in this particular example. Autofluorescence was useful as a validating method for this particular example, but it is not a universal method applicable for any mixture of microorganisms.

According to the statistical analysis, the sampling size was implied to be sufficient to represent the population, and therefore population size is equal to sampling size and population standard deviation is equal to sample standard deviation. A standardized variable [13] of 1.5 was used in setting up the relative standing of each population to obtain the optimal confidence interval as well as the confidence level between the species. The confidence level was 93.3% at a standardized variable of 1.5 from the statistic equations and the Z score Table [13]. The confidence interval of each species with different dyes was calculated. A 93% of accuracy was obtained in a case study of a two species mixed culture. The genomic copy number of slow growth pure culture can be analyzed by real time PCR method [14], [15] and it is well agreed with fluorescence-activated cell sorting (FACS) analysis or radioactive labeling genome analysis [3].

## Figures and Tables

**Table 1:** Comparison of some existing fingerprinting molecular methods and SAMI.

Methods	*In situ* (community structure remained)	Cells are kept alive	Dimensions	Relative genomic copy number	Possibility of analyzing co-culture
FISH	Y	Y	2D	N	N
DGGE	N	N	Not available	N	Y
Feulgen stain with microspectrophotometer [16]	N	N	2D	N	N
Comparative genomic hybridization [2]	N	N	Not available	Y	N
SAMI	Y	Y	3D	Y	Y
